# Recombinant human activated protein C attenuates endotoxin-induced lung injury in awake sheep

**DOI:** 10.1186/cc6985

**Published:** 2008-08-15

**Authors:** Kristine Waerhaug, Vladimir N Kuklin, Mikhail Y Kirov, Mikhail A Sovershaev, Bodil Langbakk, Ole C Ingebretsen, Kirsti Ytrehus, Lars J Bjertnaes

**Affiliations:** 1 Department of Anesthesiology, Institute of Clinical Medicine, Faculty of Medicine, University of Tromsø, Norway; 2 Department of Anesthesiology, Northern State Medical University, Arkhangelsk, Russian Federation; 3 Department of Biochemistry, Institute of Medical Biology, Faculty of Medicine, University of Tromsø, Norway; 4 Department of Medical Biochemistry, University Hospital of North Norway, Tromsø, Norway; 5 Department of Medical Biochemistry, Institute of Medical Biology, Faculty of Medicine, University of Tromsø, Norway; 6 Department of Medical Physiology, Institute of Medical Biology, Faculty of Medicine, University of Tromsø, Norway

## Abstract

**Introduction:**

Acute lung injury often complicates severe sepsis. In Gram-negative sepsis, bacterial endotoxin activates both coagulation and inflammation. Enhanced lung vascular pressures and permeability, increased extravascular lung water content and deteriorated gas exchange characterize ovine endotoxin-induced lung injury, a frequently used model of acute lung injury. Recombinant human activated protein C (rhAPC), with its anticoagulant, anti-inflammatory, fibrinolytic and antiapoptotic effects, reportedly reduces the respirator-dependent days and the mortality of patients with severe sepsis. We speculate whether rhAPC antagonizes endotoxin-induced lung injury in sheep.

**Methods:**

Two groups of sheep were exposed to *Escherichia coli *endotoxin (lipopolysaccharide) 15 ng/kg/minute intravenously from 0 to 24 hours; one group received only lipopolysaccharide throughout (n = 8), and the other group received lipopolysaccharide in combination with rhAPC 24 μg/kg/hour from 4 to 24 hours (n = 9). In addition, one group received rhAPC as above as the only intervention (n = 4), and four sham-operated sheep were used for determination of the α and ε isoforms of protein kinase C in pulmonary tissue. Data were assessed by one-way analysis of variance for repeated measurements. Biochemical data were analyzed using Student's *t *test, or using the Mann–Whitney U test when appropriate.

**Results:**

Infusion of endotoxin caused lung injury, manifested by increments in pulmonary artery pressure, in pulmonary micro-occlusion pressure, in pulmonary vascular downstream resistance, in pulmonary vascular permeability index, in extravascular lung water index and in deterioration of oxygenation that were all attenuated by infusion of rhAPC. Endotoxemia led to changes in inflammation and coagulation, including pulmonary neutrophil accumulation paralleled by increased TNFα and decreased protein C and fibrinogen in animal plasma, which all improved following infusion of rhAPC. Moreover, rhAPC prevented the translocation of protein kinase C α and ε isoforms from the cytosolic fraction of lung tissue extracts.

**Conclusion:**

In awake sheep, rhAPC alleviates endotoxin-induced lung injury – as characterized by improvements of oxygenation, coagulation and inflammation, as well as by reversal of pulmonary hemodynamic and volumetric changes.

## Introduction

Acute lung injury (ALI) is one of the most frequent organ dysfunctions evolving from severe sepsis [1]. In Gram-negative sepsis, endotoxin (lipopolysaccharide (LPS)) released from bacteria stimulates transcriptional factors to activate the inflammation and coagulation systems [2]. Proinflammatory cytokines, tissue factor, eicosanoids and endothelins act in concert to promote the disintegration of lung endothelial and epithelial barriers that characterize ALI [3-7]. Recent research has revealed that thrombin, a blood coagulation protease, is a key trigger of both the coagulation and the inflammation system in ALI [8].

Protein C is an inactive precursor of the serine protease activated protein C (APC), which is catalyzed by the action of thrombin/thrombomodulin complex when protein C is bound to its endothelial receptor (endothelial cell protein C receptor). Acting together with protein S, APC precludes generation of thrombin in a negative feedback loop by proteolytic cleavage of activated factors V and VIII. APC also escalates fibrinolysis by inhibiting plasminogen activator inhibitor 1 and thrombin-activatable fibrinolysis inhibitor [9]. Activated protein C attenuates inflammation by inhibiting translocation of NF-κB and of activator protein-1 in LPS-stimulated monocytes, thereby suppressing release of proinflammatory cytokines [10,11]. Moreover, APC decreases leukocyte rolling and adhesion to the endothelium by suppressing adhesion molecules, causing reduced migration and accumulation of leukocytes in the air spaces [10-13]. Investigators showed recently that cleavage of the protease-activated receptor-1 by the APC-endothelial cell protein C receptor complex exerts antiapoptotic and enhanced barrier-protective effects in endothelial cells via transactivation of the sphingosine-1-phosphate receptor [14,15].

In sepsis and ALI both the production and the activation of protein C decrease because of extended endothelial damage [16]. Elevation of the plasma concentration of protein C, which normally occurs upon activation of the coagulation system, is hence reversed with depletion of protein C and APC in septic states. Investigators reported recently that infusion of recombinant human activated protein C (rhAPC) enhances the plasma concentration of protein C and lowers the mortality rate of patients with severe sepsis, simultaneously reducing circulatory dysfunction and respirator-dependent days [17,18]. Although many effects of APC on coagulation and inflammation are well established [9,19], those effects dealing specifically with respiratory functions have been sparsely investigated [20,21]. Whether APC protects efficiently against ALI in human sepsis and in animals exposed to endotoxin is therefore still undetermined.

In sheep, LPS enhances lung microvascular pressure and permeability, thereby increasing the extravascular lung water content and deteriorating gas exchange [22-25], which is consistent with most signs of ALI in humans [26]. Activation of inflammatory mediators and coagulation factors accompanies the changes in pulmonary hemodynamics and gas exchange [5-7,20,24,25,27]. We speculated whether rhAPC, given its anti-inflammatory and anticoagulation properties, would antagonize these changes. Our aim was to investigate the effects of rhAPC on sustained LPS-induced ALI in sheep over 24 hours by assessing changes in pulmonary hemodynamics, in extravascular lung water content, in pulmonary vascular permeability index and in gas exchange. Moreover, we followed alterations in selected variables of coagulation and inflammation.

## Materials and methods

The Norwegian Experimental Animal Board approved the study according to the rules and regulations of the Helsinki Convention for Use and Care of Animals.

### Animal instrumentation

Twenty-five yearling sheep weighing 44.1 ± 2.5 kg (mean ± standard error of the mean) were instrumented under general anesthesia. In brief, we placed introducers in the left external jugular vein and in the ipsilateral common carotid artery, and via a left thoracotomy we inserted a medical-grade catheter into the left atrium, as described previously by our group [25]. After 1 week of recovery, the sheep were placed in an experimental pen. A flow-directed thermal dilution catheter (131HF7; Baxter, Irvine, CA, USA) was introduced into the pulmonary artery and a 4-Fr fiberoptic thermistor catheter (PV2024L; Pulsion Medical Systems, München, Germany) was introduced into the thoracic aorta. The catheters were connected to pressure transducers (Transpac III; Abbott, North Chicago, IL, USA).

### Measurements and samples

Measurements were performed at 4-hour intervals. The heart rate, mean systemic arterial pressure, mean pulmonary arterial pressure (PAP), pulmonary arterial occlusion pressure, pulmonary capillary micro-occlusion pressure (Pmo), mean left atrial pressure (LAP), and mean right atrial pressure were recorded on a Gould 6600 signal conditioner (Gould Instruments, OH, USA, USA), as previously reported [24,25,28]. The cardiac index (CI), pulmonary blood volume index, intrathoracic blood volume index, extravascular lung water index (EVLWI), and right ventricular end-diastolic volume index were determined with the thermal-dye dilution method (Cold Z-021; Pulsion Medical Systems AG). Every value was calculated as a mean of five measurements, each consisting of a 5-ml bolus of indocyanine green at 0.5 mg/ml (Pulsion Medical Systems) in ice-cold 5% glucose injected into the right atrium [24,25].

In addition, the pulmonary vascular permeability index was calculated as PVPI = EVLW/PBV [29]. The pulmonary vascular resistance index (PVRI) was calculated as PVRI = (PAP - LAP)/CI. The pulmonary vascular upstream resistance index (PVRI_up _= (PAP - Pmo)/CI) and the pulmonary vascular downstream resistance index (PVRI_dwn _= (Pmo - LAP)/CI) were also determined. The systemic vascular resistance index, the stroke volume index, the left and right ventricular stroke work indices, and the right ventricular ejection fraction were calculated using standard formulas provided by the Cold Z-021.

Blood samples were drawn from systemic and pulmonary artery lines, and were analyzed for blood gases and hemoglobin (Rapid 860; Chiron Diagnostics Corporation, East Walpole, MA, USA). The alveolar–arterial oxygen tension difference, the oxygen delivery index, the oxygen consumption index, and the venous admixture were calculated as described previously [25]. Plasma concentrations of TNF1α were determined with an Immulite instrument using a commercially available kit (Diagnostic Products Corporation, Los Angeles, CA, USA). Protein C levels and antithrombin III levels were both determined by commercially available kits using chromogenic substrates (STA-STACHROM^®^; Diagnostica Stago, Asnieres, France). The International Normalized Ratio determined factor II, factor VII and factor X combined by the measuring clotting time (prothrombin time) in the presence of tissue thromboplastin. All factors were present in excess, except for factors II, VII and X, all derived from the plasma being tested. Fibrinogen was determined with a modified Clauss method using reagents and an instrument (STA-R) from Diagnostica Stago according to the instructions of the manufacturer.

### Experimental protocol

Sheep were randomly assigned to three groups: the rhAPC group (n = 4) received an intravenous infusion of 24 μg/kg/hour drotrecogin-alpha (Xigris; Eli Lilly & Co., Indianapolis, IN, USA) from 4 to 24 hours; the LPS group (n = 8) received an intravenous infusion of *Escherichia coli *O26:B6 endotoxin (LPS) (Sigma Chemical, St Louis, MO, USA) at 15 ng/kg/minute from 0 to 24 hours; and the LPS + rhAPC group (n = 9) received LPS and rhAPC at the same doses as above. In addition, four sham-operated sheep were used only for detection of protein kinase C (PKC) in lung tissue. All sheep had an infusion of isotonic saline at 3 ml/kg/hour throughout the experiment. After completion of the experiment, euthanasia was performed with an intravenous injection of 100 mg/kg pentobarbital (Pentobarbital NAF; Ås Production Lab, Ås, Norway) followed by 50 mmol KCl. Lung biopsies from all lobes were frozen in liquid nitrogen for later determination of the α and ε isoforms of PKC by western blotting and for histological examination.

### Western blotting

The protein extraction, fractionation and detection procedure is described in detail elsewhere [24]. Briefly, lung tissue was homogenized and, after centrifugation at 100,000 × *g *for 60 minutes at 4°C, the supernatant (cytosolic fraction) was collected and used for western blotting using rabbit polyclonal antibodies against PKC isoenzymes α and ε (SC-208 and SC-214, respectively; Santa Cruz Biotech., Santa Cruz, CA, USA). Immunopositive bands were quantified by densitometry.

### Lung sampling for histologic examination

Representative tissue blocks from all lung lobes were preserved in 4% formaldehyde, were sectioned and stained with H&E, and were examined by light microscopy for evidence of lung injury. The lung injury score was assessed according to Zhou and colleagues [30]. Briefly, a pathologist without knowledge of the group identity scored each section based on the presence and the degree of edema, neutrophil sequstration, hemorrhage, epithelial desquamation and alveolar hyaline membranes, with zero as the lowest score and four as the highest score, as reported previously from our group [25]. Typical photomicrographs were taken using a Leica DM 2500 microscope and a Leica DFC 320 digital camera, with the software Leica IM50 (Leica Microsystems GmbH, Wetzlar, Germany).

### Statistical analysis

Data are expressed as the mean ± standard error of the mean. The Kolmogorov-Smirnov test was used to assess the data distribution. Normally distributed data were analyzed by two-factor analysis of variance for repeated measurements (SPSS 14.0 for Windows; LEAD Technologies, Chicago, IL, USA). To evaluate differences within groups over time, we employed test of contrasts. The Greenhouse–Geisser epsilon factor-adjusted probability levels were used when Mauchly's test was significant. For evaluation of coagulation and inflammatory markers, a *t *test was employed to test for differences between the LPS group and the LPS + rhAPC group at 24 hours. When a normal distribution of data could not be demonstrated, the Mann–Whitney U test was employed to compare the LPS and the LPS + rhAPC groups. Missing values were not replaced by imputation. *P *< 0.05 was considered statistically significant.

## Results

**Figure 1:**
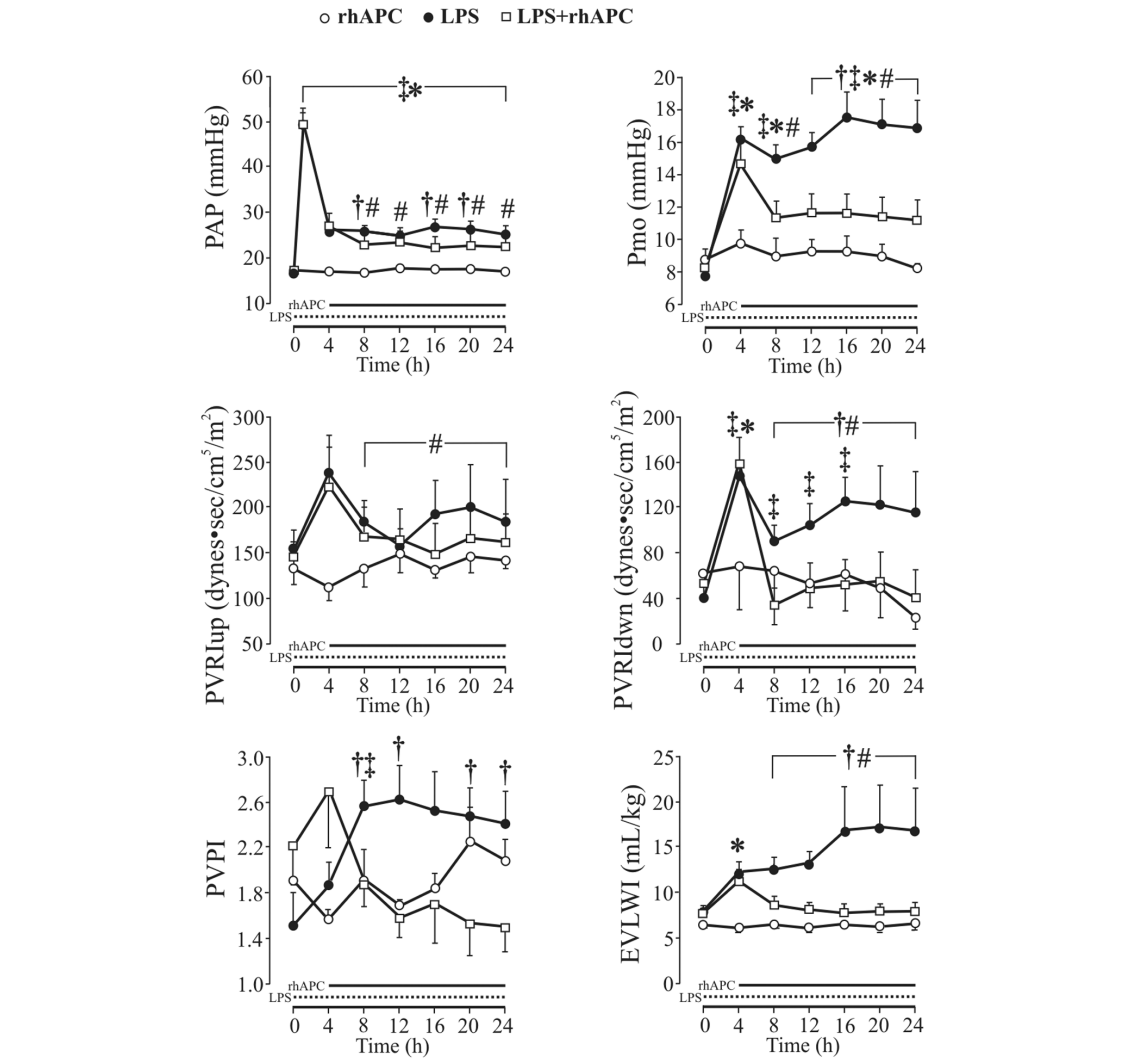
Changes in pulmonary vascular pressures and fluid filtration in awake, endotoxemic sheep. rhAPC, recombinant human activated protein C alone group (n = 4); LPS, lipopolysaccharide group (n = 8); LPS + rhAPC, rhAPC-treated LPS group (n = 9). Data presented as the mean ± standard error of the mean. PAP, pulmonary arterial pressure; Pmo, pulmonary micro-occlusion pressure; PVRIup, upstream pulmonary vascular resistance index; PVRIdwn, downstream pulmonary vascular resistance index; EVLWI, extravascular lung water index; PVPI, pulmonary vascular permeability index. †*P *< 0.05 between the LPS and the LPS + rhAPC groups; ‡*P *< 0.05 from *t *= 0 hours in the LPS group; §*P *< 0.05 from *t *= 4 hours in the LPS group; *P < 0.05 from *t *= 0 hours in the LPS + rhAPC group; #*P *< 0.05 from *t *= 4 hours in the LPS + rhAPC group.

One sheep from the LPS group died of respiratory failure after 20 hours, and one animal from the LPS + rhAPC group died of circulatory failure after 16 hours. None of the animals suffered from bleeding. As displayed in Figure 1 and Table 1, the pulmonary hemodynamic and volumetric parameters remained unchanged with rhAPC alone. The infusion of rhAPC, however, decreased the LPS-induced elevations of PAP, Pmo and PVRIdwn with intergroup differences (*P *< 0.05). The PVRI_up _and the PVRI also declined with rhAPC treatment (*P *< 0.05), but with no intergroup differences (*P *= 0.63 and P = 0.17). The pulmonary arterial occlusion pressure remained unchanged. Infusion of LPS led to rapid and sustained rises in the PVPI (*P *< 0.05) and the EVLWI (*P *< 0.001), which were both antagonized by infusion of rhAPC (*P *< 0.001) with differences between the groups (*P *< 0.01 and *P *< 0.05 for PVPI and EVLWI, respectively).

**Table 1:** Hemodynamic variables in instrumented awake sheep

Variable	Time							
	
	0 hour	1 hour	4 hours	8 hours	12 hours	16 hours	20 hours	24 hours
PVRI (dyne/s/cm^5^/m^2^)								
rhAPC	194 ± 22	192 ± 22	179 ± 38	198 ± 58	202 ± 40	193 ± 38	195 ± 33	166 ± 20
LPS	188 ± 12	810 ± 94*	359 ± 31*	252 ± 29	227 ± 21	266 ± 35	268 ± 54	248 ± 56
LPS + rhAPC	201 ± 10	847 ± 124*	392 ± 44*	215 ± 29^†^	224 ± 39^†^	209 ± 41^†^	229 ± 38^†^	210 ± 41^†^
PAOP (mmHg)								
rhAPC	8 ± 0	8 ± 0	9 ± 0	8 ± 1	10 ± 0	9 ± 1	9 ± 1	8 ± 1
LPS	8 ± 0	18 ± 1*	12 ± 0*	12 ± 1*	12 ± 1*	12 ± 1*	13 ± 1*	11 ± 0*
LPS + rhAPC	8 ± 1	15 ± 2*	12 ± 1*	11 ± 1*	10 ± 1*	11 ± 1*	11 ± 1*	10 ± 1
RAP (mmHg)								
rhAPC	3 ± 0	3 ± 1	6 ± 1*	5 ± 1*	5 ± 1	5 ± 1*	5 ± 0*	5 ± 1
LPS	4 ± 1	5 ± 1	7 ± 1*	7 ± 2	7 ± 1*	7 ± 1*	8 ± 1*	8 ± 1*
LPS + rhAPC	3 ± 1	2 ± 1	6 ± 1*	5 ± 1*	6 ± 1*	6 ± 1*	6 ± 2*	5 ± 2
Heart rate (beats/min)								
rhAPC	94 ± 8	91 ± 8	95 ± 6	92 ± 7	95 ± 4	97 ± 5	97 ± 6	92 ± 7
LPS	106 ± 4	139 ± 14	129 ± 7	151 ± 9	134 ± 8	127 ± 7	142 ± 12	137 ± 11
LPS + rhAPC	94 ± 5	158 ± 16*	112 ± 9*	151 ± 10* ^†^	138 ± 11* ^†^	128 ± 12*	122 ± 13	123 ± 12
MAP (mmHg)								
rhAPC	103 ± 3	100 ± 4	103 ± 3	106 ± 5	106 ± 2	101 ± 3	105 ± 1	100 ± 5
LPS	101 ± 3	107 ± 5	110 ± 3*	90 ± 3* ^†^	88 ± 4* ^†^	93 ± 3^†^	91 ± 3* ^†^	90 ± 3* ^†^
LPS + rhAPC	101 ± 3	105 ± 4	108 ± 2	95 ± 4	95 ± 4	96 ± 5	95 ± 4	98 ± 5
SVRI (dyne/s/cm^5^/m^2^)								
rhAPC	1677 ± 70	1688 ± 82	1610 ± 108	1877 ± 327	1918 ± 302	1680 ± 204	1872 ± 186	1671 ± 268
LPS	1587 ± 69	1976 ± 165*	1962 ± 90*	1212 ± 87* ^†^	1089 ± 82* ^†^	1278 ± 101* ^†^	1267 ± 139* ^†^	1194 ± 111* ^†^
LPS + rhAPC	1559 ± 68	1789 ± 153	1910 ± 107*	1302 ± 153^†^	1226 ± 121^†^	1268 ± 119^†^	1337 ± 141^†^	1367 ± 135^†^
Cardiac index (l/min/m^2^)								
rhAPC	5.4 ± 0.6	5.3 ± 0.7	5.2 ± 0.4	5.0 ± 0.8	5.0 ± 0.7	5.2 ± 0.6	4.9 ± 0.6	5.1 ± 0.6
LPS	5.3 ± 0.3	5.0 ± 0.7	4.3 ± 0.2*	6.0 ± 0.5^†^	6.0 ± 0.2^†^	5.7 ± 0.3^†^	5.9 ± 0.5^†^	6.0 ± 0.5^†^
LPS + rhAPC	5.1 ± 0.3	4.9 ± 0.4	4.3 ± 0.2	5.8 ± 0.3^†^	6.1 ± 0.4^†^	5.9 ± 0.4^†^	5.7 ± 0.5^†^	5.8 ± 0.5^†^
SVI (ml/beat/m^2^)								
rhAPC	57 ± 3	57 ± 2	55 ± 2	54 ± 6	52 ± 7	55 ± 7	52 ± 8	57 ± 8
LPS	49 ± 2	36 ± 2*	33 ± 2*	40 ± 4	46 ± 3	45 ± 2	44 ± 5	46 ± 4
LPS + rhAPC	55 ± 2	33 ± 4*	40 ± 3*	39 ± 3*	46 ± 4*	49 ± 5	50^† ^± 5	49 ± 5
LAP (mmHg)								
rhAPC	5 ± 1	5 ± 1	6 ± 2	6 ± 1	6 ± 1	6 ± 1	6 ± 1	7 ± 1
LPS	5 ± 1	5 ± 1	8 ± 1*	8 ± 1*	8 ± 1*	9 ± 1*	9 ± 1*	9 ± 1*
LPS + rhAPC	5 ± 1	3 ± 1	6 ± 1	9 ± 2*	8 ± 2*	9 ± 2*	8 ± 2*	9 ± 2*
LVSWI (g/m/m^2^)								
rhAPC	67 ± 3	69 ± 4	68 ± 3	69 ± 12	62 ± 8	63 ± 11	61 ± 12	64 ± 11
LPS	62 ± 3	42 ± 3*	44 ± 2*	43 ± 4*	49 ± 5	50 ± 3*	47 ± 6*	49 ± 5*
LPS + rhAPC	69 ± 4	42 ± 6*	52 ± 4*	44 ± 3*	53 ± 4*	56 ± 5*	56 ± 5*	59 ± 7
RVSWI (g/m/m^2^)								
rhAPC	11 ± 1	11 ± 1	9 ± 1	9 ± 1	9 ± 1	9 ± 1	9 ± 1	10 ± 2
LPS	9 ± 0	22 ± 1*	9 ± 0	11 ± 1	12 ± 1	12 ± 1*	11 ± 1	10 ± 1
LPS + rhAPC	10 ± 1	21 ± 2*	11 ± 1	9 ± 1	11 ± 1	10 ± 1	11 ± 2	11 ± 2

Data presented as the mean ± standard error of the mean. rhAPC, recombinant human activated protein C alone (n = 4); LPS, lipopolysaccharide group (n = 8); LPS + rhAPC, rhAPC-treated LPS group (n = 9); PVRI, pulmonary vascular resistance index; PAOP, pulmonary arterial occlusion pressure; RAP, right atrial pressure; MAP, mean arterial pressure; SVRI, systemic vascular resistance index; SVI, stroke volume index; LAP, left atrial pressure; LVSWI, left ventricle stroke work index; RVSWI, right ventricle stroke work index. *P < 0.05 from *t *= 0 hours. ^†^*P *< 0.05 from *t *= 4 hours.

The intrathoracic blood volume index, pulmonary blood volume index, right ventricle end-diastolic volume and right ventricle ejection fraction did not change significantly between the groups (data not shown). Moreover, infusion of rhAPC had no significant effects on the LPS-induced changes in right atrial pressure, heart rate, mean systemic arterial pressure, systemic vascular resistance index, CI, stroke volume index, LAP, and left and right ventricle stroke work indices in any of the groups (Table 1).

**Figure 2:**
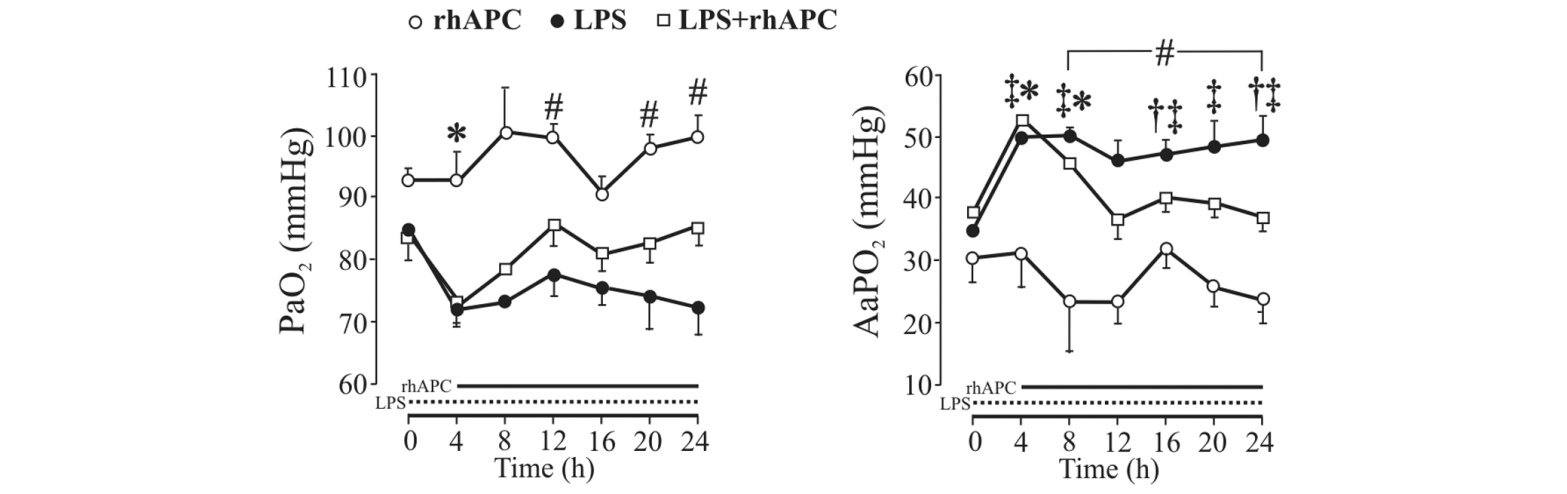
Changes in arterial oxygenation in awake, endotoxemic sheep. rhAPC, recombinant human activated protein C alone group (n = 4); LPS, lipopolysaccharide group (n = 8); LPS + rhAPC, rhAPC-treated LPS group (n = 9). Data presented as the mean ± standard error of the mean. PaO_2_, arterial oxygen partial pressure; AaPO_2_, alveolar–arterial oxygen tension difference. †*P *< 0.05 between the LPS and the LPS + rhAPC groups; ‡*P *< 0.05 from *t *= 0 hours in the LPS group; §*P *< 0.05 from *t *= 4 hours in the LPS group; *P < 0.05 from *t *= 0 hours in the LPS + rhAPC group; #*P *< 0.05 from *t *= 4 hours in the LPS + rhAPC group.

Figure 2 and Table 2 show no changes in oxygenation after rhAPC alone. Infusion of LPS decreased the PaO_2 _abruptly in concert with a rise in the alveolar–arterial oxygen tension difference (*P *< 0.01), which improved significantly in response to administration of rhAPC (*P *< 0.05). Mixed venous oxygen saturation and PaCO_2 _increased in the LPS + rhAPC group (*P *< 0.05), but with no significant intergroup differences. Exposure to LPS increased the body temperature and oxygen delivery index (*P *< 0.001), and tended to increase the oxygen consumption index (*P *= 0.051). Treatment with rhAPC, however, had no significant effect on these changes. The hemoglobin concentration initially increased during LPS infusion (*P *< 0.001) and decreased during rhAPC treatment (*P *< 0.001), but with no significant intergroup difference.

**Table 2:** Respiratory and metabolic variables in instrumented awake sheep

	Time						
	
	0 hours	4 hours	8 hours	12 hours	16 hours	20 hours	24 hours
SaO_2 _(%)							
rhAPC	96 ± 3	95 ± 3	98 ± 1	99 ± 1	98 ± 1	98 ± 1	99 ± 1
LPS	98 ± 0	98 ± 1	98 ± 1	98 ± 1	98 ± 1	97 ± 2	97 ± 2
LPS + rhAPC	98 ± 0	96 ± 1	98 ± 0	98 ± 1	97 ± 1	97 ± 1	98 ± 1
SvO_2 _(%)							
rhAPC	61 ± 4	59 ± 4	61 ± 5	59 ± 5	61 ± 4	61 ± 5	63 ± 4
LPS	62 ± 3	62 ± 3	69 ± 2* ^†^	67 ± 3*	64 ± 3	63 ± 3	61 ± 3
LPS + rhAPC	63 ± 3	61 ± 3	70 ± 3^†^	66 ± 2^†^	67 ± 2* ^†^	66 ± 2	67 ± 2* ^†^
DO_2_I (ml/min/m^2^)							
rhAPC	640 ± 64	649 ± 7	626 ± 85	573 ± 63	614 ± 39	595 ± 72	654 ± 54
LPS	608 ± 32	585 ± 48	785 ± 53*	763 ± 62*	731 ± 45*	748 ± 70*	751 ± 73*
LPS + rhAPC	642 ± 52	611 ± 38	802 ± 76* ^†^	772 ± 58^†^	732 ± 51^†^	693 ± 51	704 ± 55
VO_2_I (ml^/^min/m^2^)							
rhAPC	225 ± 37	244 ± 26	243 ± 63	222 ± 46	215 ± 28	206 ± 12	218 ± 28
LPS	226 ± 7	207 ± 11	232 ± 15	234 ± 14	252 ± 19	261 ± 24	277 ± 30
LPS + rhAPC	229 ± 26	231 ± 22	225 ± 21	249 ± 20	232 ± 17	227 ± 19	227 ± 22
Qs/Qt							
rhAPC	0.12 ± 0.06	0.13 ± 0.06	0.05 ± 0.02	0.04 ± 0.02	0.08 ± 0.02	0.04 ± 0.01	0.02 ± 0.01
LPS	0.05 ± 0.01	0.11 ± 0.03	0.13 ± 0.03	0.13 ± 0.04	0.12 ± 0.04	0.10 ± 0.04	0.10 ± 0.04
LPS + rhAPC	0.07 ± 0.01	0.11 ± 0.02	0.10 ± 0.01	0.08 ± 0.03	0.10 ± 0.03	0.09 ± 0.03	0.08 ± 0.03
Arterial pH							
rhAPC	7.50 ± 0.01	7.47 ± 0.01	7.44 ± 0.03	7.44 ± 0.04	7.45 ± 0.03	7.49 ± 0.02	7.48 ± 0.02
LPS	7.51 ± 0.01	7.55 ± 0.01	7.54 ± 0.01	7.54 ± 0.01	7.52 ± 0.03	7.53 ± 0.03	7.53 ± 0.03
LPS + rhAPC	7.49 ± 0.02	7.54 ± 0.01*	7.51 ± 0.01	7.49 ± 0.01^†^	7.50 ± 0.01^†^	7.52 ± 0.01* ^†^	7.51 ± 0.01^†^
PaCO_2 _(kPa)							
rhAPC	33 ± 2	33 ± 1	33 ± 1	34 ± 2	35 ± 1	33 ± 1	33 ± 1
LPS	36 ± 1	35 ± 3	33 ± 1	33 ± 1	34 ± 1	34 ± 1	35 ± 1
LPS + rhAPC	36 ± 1	31 ± 2*	32 ± 1*	34 ± 2	36 ± 2^†^	35 ± 2^†^	35 ± 2^†^
Hemoglobin (g/dl)							
rhAPC	9.1 ± 1.2	9.1 ± 1.3	9.2 ± 1.2	8.7 ± 1.0	8.9 ± 1.1	9.2 ± 1.1	9.3 ± 1.1
LPS	8.7 ± 0.7	10.4 ± 0.6*	10,3 ± 0.7*	9.6 ± 0.6* ^†^	9.7 ± 0.7*	9.7 ± 0.6*	9.6 ± 0.7*
LPS + rhAPC	9.5 ± 0.6	10.8 ± 0.6*	10.2 ± 0.6^†^	9.6 ± 0.6^†^	9.4 ± 0.5^†^	9.3 ± 0.4^†^	9.4 ± 0.5^†^
Temperature (°C)							
rhAPC	39.4 ± 0.1	39.4 ± 0.1	39.3 ± 0.2	39.4 ± 0.2	39.3 ± 0.2	39.2 ± 0.2	39.1 ± 0.1
LPS	39.2 ± 0.2	40.8 ± 0.3*	40.2 ± 0.2*	40.3 ± 0.2*	40.5 ± 0.2*	40.3 ± 0.3*	40.1 ± 0.2*
LPS + rhAPC	39.1 ± 0.1	41.0 ± 0.3*	40.2 ± 0.2* ^†^	40.0 ± 0.1* ^†^	40.0 ± 0.1* ^†^	40.0 ± 0.2* ^†^	39.9 ± 0.2* ^†^
Antithrombin III plasma concentration (%)							
rhAPC	70 ± 2	74 ± 2		70 ± 2			70 ± 3
LPS	79 ± 3	71 ± 4*		66 ± 4* ^†^			63 ± 4* ^†^
LPS + rhAPC	69 ± 3	69 ± 3		62 ± 3* ^†^			58 ± 2* ^†^

Data presented as the mean ± standard error of the mean. rhAPC, recombinant human activated protein C alone group (n = 4); LPS, lipopolysaccharide group (n = 8); LPS + rhAPC, rhAPC-treated LPS group (n = 9). SaO_2_, arterial oxygen saturation; SvO_2_, central venous oxygen saturation; DO_2_I, oxygen delivery index; VO_2_I, oxygen consumption index; Qs/Qt, venous admixture. *P < 0.05 from *t *= 0 hours. ^†^*P *< 0.05 from *t *= 4 hours.

**Figure 3:**
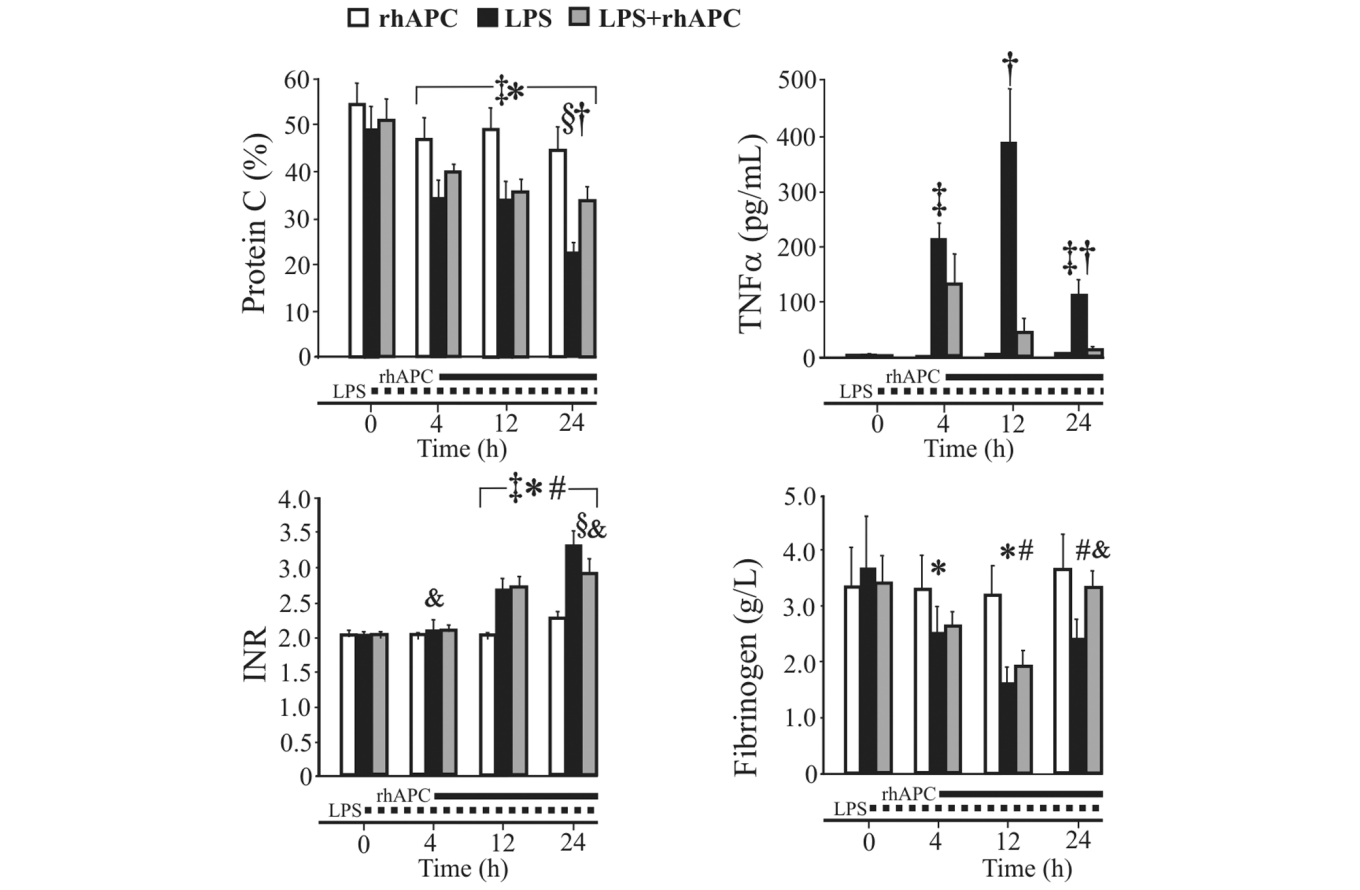
Changes in coagulation and inflammatory markers in awake, endotoxemic sheep. rhAPC, recombinant human activated protein C alone group (n = 4); LPS, lipopolysaccharide group (n = 8); LPS + rhAPC, rhAPC-treated LPS group (n = 9). Data presented as the mean ± standard error of the mean. INR, International Normalized Ratio. †*P *< 0.05 between the LPS and the LPS + rhAPC groups; ‡*P *< 0.05 from *t *= 0 hours in the LPS group; §*P *< 0.05 from *t *= 4 hours in the LPS group; *P < 0.05 from *t *= 0 hours in the LPS + rhAPC group; #*P *< 0.05 from *t *= 4 hours in the LPS + rhAPC group; &*P *< 0.05 from *t *= 0 hours in the rhAPC group.

With rhAPC alone, the protein C and antithrombin III levels remained unchanged while the International Normalized Ratio and the fibrinogen level increased by nearly 10% (*P *< 0.05; Table 2 and Figure 3). Infusion of LPS reduced protein C by 49% and 33% in the LPS group and in the LPS + rhAPC group, respectively (*P *< 0.001), with an intergroup difference at 24 hours (*P *< 0.05). In both LPS groups, fibrinogen decreased to a nadir approximately 30% below baseline and then increased in rhAPC-treated animals (*P *< 0.05), but with no difference between groups (*P *= 0.08). The antithrombin III concentration fell and the International Normalized Ratio increased gradually (*P *< 0.001), with no significant intergroup difference.

As depicted in Figure 3, TNFα peaked at 12 hours (*P *< 0.05) in the LPS group and subsequently declined to significantly above baseline at 24 hours. In the LPS + rhAPC group, TNFα decreased beyond 4 hours, with an intergroup difference after 12 hours (*P *< 0.05).

**Figure 4:**
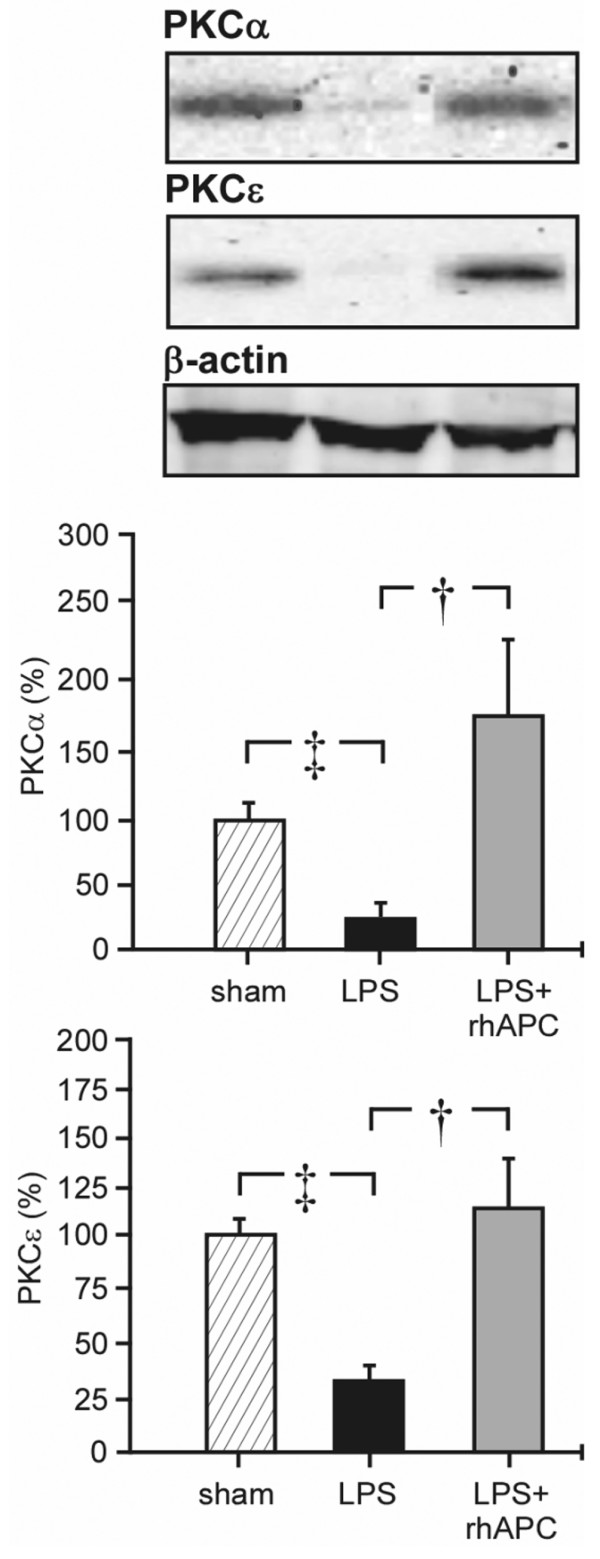
Protein kinase C α and ε isoforms in the cytosole fraction of lungs from sheep. Western blots and densitometry tracings of protein kinase C (PKC)α and PKCε in the cytosole fraction of lungs from sheep. Sham, sham-operated group (n = 4); LPS, lipopolysaccharide group (n = 8); LPS + rhAPC, rhAPC-treated LPS group (n = 9). †*P *< 0.05 between the LPS and the LPS + rhAPC groups; ‡*P *< 0.05 between the LPS and the sham groups. Data presented as the percentage of the mean densitometry value in sham-operated sheep (n = 4).

In lung tissue from sheep exposed to LPS, we found a 65% to 75% reduction of the α and ε isoforms of PKC in the cytosolic fraction of tissue homogenate, as compared with samples from sham-operated animals (*P *< 0.05; Figure 4). In the LPS + rhAPC group, the cytosolic fraction of PKCα and PKCε remained significantly higher than in the LPS group (*P *< 0.05).

**Figure 5:**
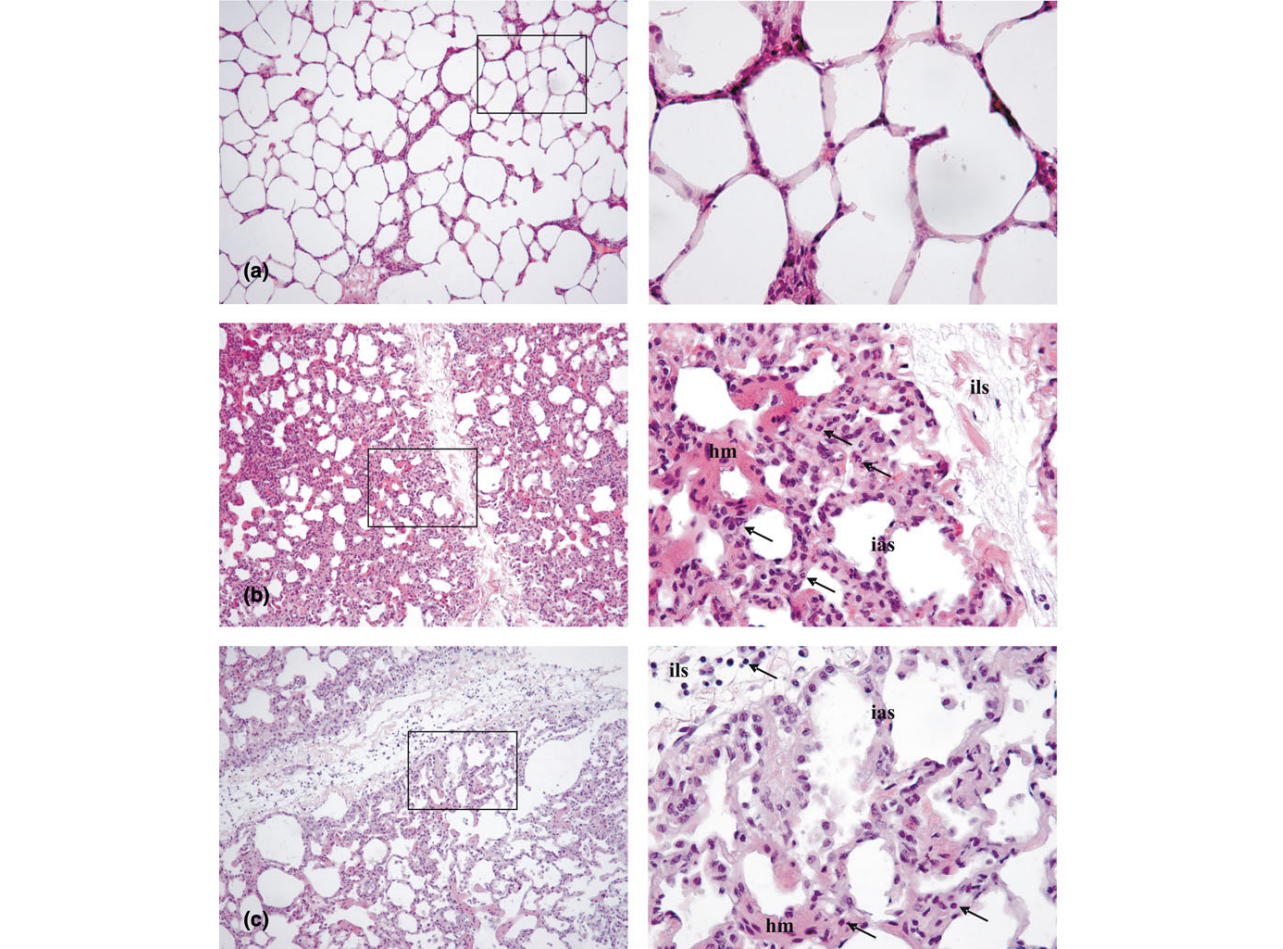
Photomicrographs of H&E-stained ovine right lung lower lobe sections from experiments over 24 hours. Left: sections magnified ×100. Right: fields limited by squares are magnified ×400. **(a) **Sheep exposed to recombinant human activated protein C (rhAPC) alone, displaying no gross changes from normal. **(b) **Sheep exposed to intravenous infusion of lipopolysaccharide (LPS). Hyperemia and intravascular accumulation and extravasation of neutrophils (arrows) are accompanied by interstitial edema and thickening of interalveolar and interlobular (ias and ils) septa as well as injury to the alveolar lining with formation of hyaline membranes (hm). **(c) **Sheep treated with infusion of rhAPC from 4 hours after the commencement of LPS. Interlobular and interalveolar septa are predominantly infiltrated with lymphocytes (arrows) and interalveolar septa have returned to nearly normal thickness. Hyaline membranes are partly dissolved.

Table 3 presents the individual scores constituting the lung injury score. In animals exposed to LPS the lung injury score was elevated (*P *< 0.01), but the score was 34% lower in the LPS + rhAPC group (*P *< 0.01). Figure 5 shows photomicrographs of typical lung sections, with low-power fields to the left. The marked squares are displayed as high-power fields to the right. In sheep subjected to rhAPC alone (Figure 5a), nearly normal histological conditions were found. Lungs exposed to LPS (Figure 5b) displayed edematous, expanded and disrupted alveoli with interstitial neutrophile infiltration. All the latter changes were less pronounced in the LPS + rhAPC group (Figure 5c).

**Table 3:** Lung injury score in instrumented awake sheep

Variable	Edema	Neutrophil infiltration	Hemorrhage	Epithelial desquamation	Hyaline membranes	Lung injury score
rhAPC	1.3 (1.0 to 2.0)	0.5 (0.0 to 2.0)	1.0 (0.0 to 3.0)	0.5 (0.0 to 1.0)	0.0 (0.0 to 1.0)	3.8 (2.0 to 6.5)
LPS	2.0 (1.0 to 3.0)	2.5 (0.5 to 3.5)*	0.8 (0.0 to 2.0)	1.0 (0.5 to 4.0)*	1.0 (0.0 to 2.0)*	6.8 (2.5 to 11.5)*
LPS + rhAPC	1.0 (0.5 to 2.0)^†^	1.0 (0.0 to 2.0)^†^	1.0 (0.0 to 2.0)	1.0 (0.5 to 2.0)	0.5 (0.0 to 2.0)	4.5 (1.5 to 8.5)^†^

Data presented as the median (minimum to maximum). rhAPC, recombinant human activated protein C alone group (n = 4); LPS, lipopolysaccharide group (n = 8); LPS + rhAPC, rhAPC-treated LPS group (n = 9). The histological markers of lung injury are scored on a scale from zero to four. *P < 0.05 between the LPS and the rhAPC groups. ^†^*P *< 0.05 between the LPS and the LPS + rhAPC groups.

## Discussion

The present study demonstrates that continuously infused rhAPC alleviates endotoxin-induced lung injury in awake sheep. The enhanced EVLWI, a major characteristic of this model, decreased significantly upon administration of rhAPC. Dampening of pulmonary vascular pressures in parallel with a reduction of PVRI_dwn _and improvement of gas exchange concerted the reduction of EVLWI. Administration of rhAPC also prevented elevation of the plasma concentration of TNFα, and normalized the plasma level of protein C. Moreover, rhAPC reduced the PVPI and counteracted the depletion of the α and ε isoforms of PKC from the cytosolic fraction of lung tissue homogenate, simultaneously maintaining pulmonary histological integrity.

Infusion of LPS provides a possibility of mimicking human ALI in different species, including sheep [22,23]. The fact that two animals died during the course of the 24-hour experiments illustrates the severity of this lung injury model, which is associated with a lethality of nearly 30% without supportive therapy [7,25]. The LPS-induced increase in the EVLWI and the changes in lung hemodynamics are consistent with observations made in previous studies employing the same dose of endotoxin over 24 hours [24,25]. Despite the fact that we treated sheep with rhAPC specifically designed for the human, it markedly reversed the LPS-induced procoagulant and proinflammatory states. This might indicate a degree of cross-reaction between the inflammation and the coagulation systems of the two species, in agreement with other recent investigations [20,21].

Following administration of rhAPC dosed as recommended for patients with severe sepsis [17], we noticed that the enhanced EVLWI declined towards baseline immediately after commencement of the drug. Furthermore, the PAP, Pmo and PVRI_dwn_, all rising during infusion of LPS, decreased markedly during rhAPC infusion. We believe that the reduction of PVRI_dwn _prompted the falls in PAP and Pmo, and therefore partly explains the rhAPC-induced reduction of EVLWI. The decrease in PAP is consistent with recent findings of Maybauer and colleagues, reporting that rhAPC, administered 1 hour subsequent to smoke inhalation and sepsis, improves pulmonary function in sheep [20]. Correspondingly, Wang and colleagues, also using an ovine model of severe sepsis, noticed that rhAPC antagonizes lactic acidosis, derangements of hemodynamics and gas exchange as well as coagulation abnormalities 2 hours subsequent to peritoneal instillation of feces [21]. The latter investigators also observed that rhAPC reduces the lung wet/dry weight ratio.

In the present study, we display the course of changes in EVLWI over 24 hours. Not obvious to us, however, was whether the decrease in EVLWI was a result of reduced Pmo, of reduced permeability, or of both reductions combined. Relating EVLWI to the lung blood volume, the PVPI supposedly indicates evolvement of edema due to increased leakiness of the lung vasculature [29,31,32]. We observed that PVPI increased and stabilized at a ratio of nearly 2.5 in the LPS group (Figure 1), which is slightly higher than that noticed by Kuzkov and colleagues in nonsurvivors of severe sepsis and ALI [31]. Moreover, the ratio of approximately 1.5 that we observed in the LPS + rhAPC group was not significantly different from baseline. Although the PVPI is not unequivocally accepted as a measure of vascular permeability [33], this finding could indicate an APC-dependent preservation of vascular integrity.

Deterioration of gas exchange accompanied the accumulation of extravascular lung water, as shown in previous investigations of ALI after LPS treatment in sheep [5,23,34]. The impaired oxygenation (Figure 2) improved significantly in the LPS + rhAPC group in parallel with a normalization of PaCO_2_, consistent with recent animal reports [20,21] and with the PROWESS study [17]. The latter workers demonstrated faster resolution of cardiovascular and respiratory dysfunctions in rhAPC-treated patients [18].

In the present investigation, administration of LPS led to depletion of protein C and fibrinogen. The increase in the plasma concentration of fibrinogen during treatment with rhAPC is consistent with the findings of Taylor and colleagues, who concluded that APC prevents both the coagulopathy and the lethal effects of infusion of live *E. coli *in baboons [19]. Moreover, the better maintained concentration of protein C following administration of rhAPC agrees with corresponding findings of Wang and colleagues in septic sheep treated with rhAPC [21]. According to a recent investigation, a decrease in protein C is a regular finding in patients with severe sepsis and an early and independent predictor of sepsis mortality [35].

In the present study, the enhanced plasma concentration of TNFα and the depletion of PKCα and PKCε from the cytosolic fraction of lung tissue homogenate after LPS, as previously reported [24], vanished during treatment with rhAPC. Moreover, histological examination revealed less inflammatory changes in rhAPC-treated endotoxemic animals, as evaluated by reduced neutrophil infiltration and decreased edematous changes in lung tissue. Our results agree with an investigation in endotoxemic rats demonstrating an APC-induced decrease in the lung wet/dry weight ratio in parallel with reduced accumulation of leukocytes in the lungs and a fall in the plasma concentration of TNFα [12,36]. In volunteers subjected to instillation of LPS into the airways, Nick and colleagues demonstrated increased neutrophil migration into the air spaces that was prevented with rhAPC [13]. The latter observation is consistent with our observation that the lung injury score decreased mainly as a result of less neutrophils in the lung sections of rhAPC-treated endotoxemic sheep. Moreover, Kirschenbaum and colleagues noticed that rhAPC prevents neutrophil-platelet interaction with endothelial cells from healthy subjects, when stimulated with plasma from patients in septic shock. These authors suggested that rhAPC preserves microvascular patency in severe sepsis [37], which is consistent with the regression of LPS-induced increases in the PVPI and the EVLWI after infusion of rhAPC in the present study. In this study, plasma concentrations of TNFα peaked 12 hours after administration of LPS. The sustained high levels of TNFα in the LPS group is consistent with observations in patients with sepsis, in whom persistent high levels of TNFα correlates with severity of illness [38].

Some limitations must be taken into account when evaluating the results of this study. Firstly, coagulation and inflammatory markers have in total 17% missing values that reduce the power of the study as these variables are concerned. Secondly, we used human kits for determination of TNFα and for coagulation tests, due to the lack of kits specifically designed for sheep. As evolutionary old systems, however, both the innate immune system and the coagulation system expectedly have a high degree of cross-reactivity in mammals [39]. The change in plasma concentration of TNFα during exposure to LPS is consistent with previously reported observations from our group where we used human antibodies in combination with the same automated system for cytokine analysis [24].

## Conclusion

The present study demonstrates that infused rhAPC ameliorates ovine LPS-induced ALI, as indicated by a decrease in the EVLWI associated with improvements of gas exchange and markers of inflammation and coagulation. We suggest that treatment with rhAPC reduces lung fluid extravasation by dampening both the pressure and the permeability of the lung microvasculature. Further studies are warranted to determine whether these beneficial effects also apply in patients suffering from sepsis – and in patients from nonsepsis-induced ALI.

## Key messages

• In ovine endotoxin-induced lung injury, rhAPC counteracts the increments in pulmonary vascular downstream resistance and pulmonary vascular pressures.

• rhAPC dampens inflammation and the pulmonary vascular permeability index, antagonizing the enhanced extravascular lung water and the deranged arterial oxygenation.

## Abbreviations

AaO_2 _= alveolar-arterial oxygen tension difference; ALI = acute lung injury; APC = activated protein C; CI = cardiac index; EVLWI = extravascular lung water index; H&E = hematoxylin and eosin; LAP = left atrial pressure; LPS = lipopolysaccharide; NF = nuclear factor; PaCO_2 _= partial tension of carbon dioxide in arterial blood; PaO_2 _= partial tension of oxygen in arterial blood; PAP = pulmonary arterial pressure; PKC = protein kinase C; Pmo = pulmonary capillary micro-occlusion pressure; PVPI = pulmonary vascular permeability index; PVRI = pulmonary vascular resistance index; PVRI_dwn _= pulmonary vascular downstream resistance index; PVRI_up _= pulmonary vascular upstream resistance index; rhAPC = recombinant human activated protein C; TNF = tumor necrosis factor.

## Competing interests

The present study was supported by Helse Nord (Project Number 4001.721.477) and by the Department of Anesthesiology, University Hospital of North Norway, Tromsø, Norway, by the Department of Anesthesiology, Institute of Clinical Medicine, University of Tromsø, Norway, and in part by Eli Lilly & Co., USA. The support from Eli Lilly & Co. was limited to a reimbursement of our expenses for experimental animals and free use of the Xigris^® ^(rhAPC) that was used in the study. MYK is a member of the Advisory Board of Pulsion Medical Systems (München, Germany).

## Authors' contributions

KW analyzed the data and participated in the experiments and in writing the manuscript. VNK and MYK participated in the design of the study, in experimentation and in data analysis. MAS, BL, OCI and KY contributed with biochemical analyses and suggested textual improvements. LJB participated in the administration and design of the study, and drafted the manuscript. All authors have read and approved the final manuscript.
